# Statins Have No Additional Benefit for Pulmonary Hypertension: A Meta-Analysis of Randomized Controlled Trials

**DOI:** 10.1371/journal.pone.0168101

**Published:** 2016-12-19

**Authors:** Lin Wang, Moying Qu, Yao Chen, Yaxiong Zhou, Zhi Wan

**Affiliations:** 1 Department of Cardiology, Chengdu Shang Jin Nan Fu Hospital, Chengdu, Sichuan Province, China; 2 Department of Cardiology, West China hospital of Sichuan University, Chengdu, Sichuan Province, China; 3 Department of Emergency, West China hospital of Sichuan University, Chengdu, Sichuan Province, China; University of Manitoba, CANADA

## Abstract

**Objectives:**

We performed a meta-analysis to explore the effects of adding statins to standard treatment on adult patients of pulmonary hypertension (PH).

**Methods:**

A systematic search up to December, 2015 of Medline, EMBASE, Cochrane Database of Systematic reviews and Cochrane Central Register of Controlled Trials was performed to identify randomized controlled trials with PH patients treated with statins.

**Results:**

Five studies involving 425 patients were included into this meta-analysis. The results of our analysis showed that the statins can’t significantly increase 6-minute walking distance (6MWD, mean difference [MD] = -0.33 [CI: -18.25 to 17.59]), decrease the BORG dyspnea score (MD = -0.72 [CI: -2.28 to 0.85]), the clinical worsening risk (11% in statins vs. 10.1% in controls, Risk ratio = 1.06 [CI: 0.61, 1.83]), or the systolic pulmonary arterial pressure (SPAP) (MD = -0.72 [CI: -2.28 to 0.85]). Subgroup analysis for PH due to COPD or non-COPD also showed no significance.

**Conclusions:**

Statins have no additional beneficial effect on standard therapy for PH, but the results from subgroup of PH due to COPD seem intriguing and further study with larger sample size and longer follow-up is suggested.

## Introduction

Pulmonary hypertension (PH) is a kind of heterogeneous and progressive disorder with high morbidity and mortality, characterized by a persistent increase of pulmonary arterial resistance and subsequent right heart failure caused by vascular obstruction and restriction. According to the leading predisposing cause, PH is classified into five groups: group 1) pulmonary arterial hypertension; group 2) pulmonary hypertension due to left heart disease; group 3) pulmonary hypertension due to chronic lung disease and/or hypoxia; group 4) chronic thromboembolic pulmonary hypertension; and group 5) pulmonary hypertension due to unclear multifactorial mechanisms [1].

The current treatment to PH may include two sections: 1) general measures and supporting therapy, such as rehabilitation, exercise training, chronic calcium channel blockers, anticoagulants, diuretics, digitalis and oxygen, etc.; 2) target therapy for PH, such as endothelin receptor antagonists, nitric oxide, prostacyclin analogues, elastase inhibitors, and phosphodiesterase-5 (PDE-5) inhibitors. There are also some experimental treatment approaches as the last choice (e.g. gene therapy and lung transplantation) [2, 3]. Because of the relatively high expense and disappointing effectiveness of the above treatments, investigators began to search the old therapeutic targets for potential additional treatment for PH [3, 4].

Statins are one of these old drugs being examined and have been believed to be hopeful additional treatment by cell and animal models and some small observational studies. Statins are usually used to lower the level of cholesterols, but they have shown other cholesterol-independent biologic effects which may be helpful for PH. Statins can enhance the ability of endothelial nitric oxide synthase (eNOS) to produce nitric oxide, resulting from the direct up-regulation of eNOS mRNA [5]. RhoA/Rho-kinase signaling pathway is vital for cell proliferation, and statins can regulate this pathway, thus inhibit the proliferation and induce the apoptosis of vascular smooth muscle [6–8]. In several studies of animal models, the results have shown that statins are able to prevent or even reverse PH [8–11]. A few human studies, observational or randomized, have tested the impact of statins therapy on patients with PH, with discrepant results [12–20]. Therefore, we performed this meta-analysis to explore the effectiveness of statins added to standard therapy on pulmonary hypertension patients.

## Methods

We followed the Preferred Reporting Items for Systematic Reviews and Meta-analyses (PRISMA) guidelines [21].

### Data source and searches

An up-to-date systematic search of Medline, EMBASE, Cochrane Database of Systematic reviews and Cochrane Central Register of Controlled Trials was carried out, and the last search was on December 30, 2015. The MESH terms and text key words as following were used in various combinations, “statin”, “HMG-CoA reductase inhibitor”, “HMG-CoA RI”, “fluvastatin”, “pravastatin”, “simvastatin”, “atorvastatin”, “lovastatin”, “cerivastatin”, and “rosuvastatin” combined with “pulmonary hypertension” or “pulmonary arterial hypertension” using the Boolean operator “AND”. No limits were exerted on subjects or languages. The bibliographies of the included and relevant articles and reviews were manually searched to identify additional trials. We also browsed following websites to locate pertinent oral presentations and trials in process: AHA (http://www.aha.org), ATS (http://www.thoracic.org/), ERS (http://www.ersnet.org/) and ClinicalTrials (http://www.clinicaltrials.gov). All abstracts or manuscripts of potentially relevant articles were reviewed independently by 3 investigators (L.W, MY.Q, and YX.Z.).

### Studies Selection and data collection

**Fig 1:**
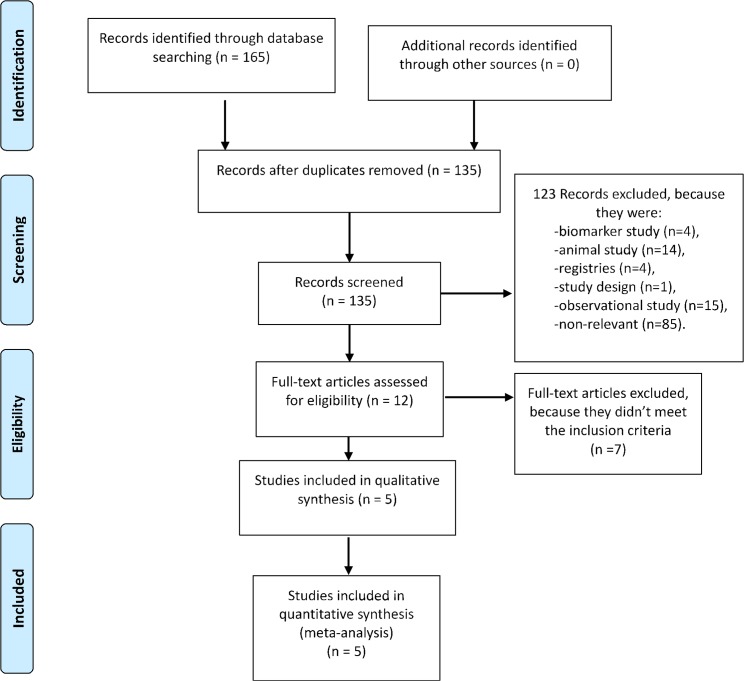
Flow chart describing systematic research and study selection process

Studies which meet the following criteria were included in this meta-analysis: 1) human subjects with pulmonary hypertension, 2) randomized trials, 3) treated with statins plus standard therapy, with standard therapy alone as control, (4) have a mean duration of follow-up of at least 24 weeks, 5) reported clinical relevant endpoints other than biomarkers. The steps of the literature search process and studies selection are outlined in Fig 1.

#### Validity Assessment

The risks of bias were assessed by 3 independent reviewers (W.L., Q.M.Y., and Z.Y.X.), regarding to: 1) randomization, 2) allocation concealment, 3) masking of treatment allocation and blinding, and 4) withdrawals. Any disagreement was settled by consensus.

Data extracted directly from the included article(s) were 1) baseline patient characteristics (age, gender and classification of PH), 2) the inclusion and exclusion criteria, 3) medication and dose for treatment and control groups, and 4) efficacy of intervention on pulmonary hypertension. For studies with insufficient data, authors were contacted via e-mail.

### Outcome and statistical analysis

Our primary outcome was the change of 6-min walk distance (6MWD) from baseline to week 24 [22, 23]. The following were used as secondary end-points: change of systolic pulmonary arterial pressure from baseline to week 24, Borg dyspnea score (score 0 for no dyspnea and 10 for maximal dyspnea) [22], and risk of clinical worsening (defined as death, the first hospitalization for PH after enrollment or initiation of PH targeted therapy).

As for subgroup analysis, because the number of PH patients from each causes mentioned above is too small, we pre-designed to divide all the patients into two subgroups according to the underlying causes: COPD and non-COPD.

This meta-analysis was done with Review Manager Software (version 5.3 for Windows; The Cochrane Collaboration, 2014). Risk ratio (RR) or mean difference (MD), with 95% confidence interval (CI) were used as summary statistics for all outcomes. Cochrane Q-test (significant at P < 0.1) and the I^2^ value were used to examine statistical heterogeneity among studies, and heterogeneity was considered present among the studies with an I^2^ > 30%. A random effects model [DerSimonian] was adopted for each outcome even with little or no evidence of heterogeneity, because many authors recommended to use random effects models in medical decision-making contexts. When assessment of the influences of individual studies on the pooled effect was necessary, sensitivity analyses were conducted by withdrawing trials one by one. P value of less than 0.05 was considered statistically significant.

## Results

### Study characteristics

Although the 5 studies involving 425 patients tried to assess similar efficacy end points and outcomes [12–14, 17, 19], they were slightly different in terms of overall design and outcome definitions (baseline characteristics of the included studies and comparison of end points across the studies, Table 1).

**Table 1:** Characteristics of included trials.

Study number		1	2	3	4	5
Author (year)		Moosavi,2013	Zeng, 2012	Kawut, 2011	Willkins,2010	Lee, 2009
Type of study		RCT	RCT	RCT	RCT	RCT
Patients stains n/Controls n		24/21	112/108	32/33	19/23	27/26
Gender (M/F)	Statins	15/9	33/79	6/26	2/17	20/7
	Controls	13/8	43/65	3/30	8/15	19/7
Mean age (year±SD)	Statins	65±11	35±13	50.0±14.3	43.2±15.2	71±8
	Controls	68±14	37±13	51.0±13.6	49.1±14.9	72±6
Cause of PH		COPD	Group 1 PH or due to inoperable CTEPH.	Group 1 PH	Group 1 PH	COPD
Intervention and dosing(mg)		Atorvastatin, 20 BID	Atorvastatin, 10 QD	Simvastatin, 40 QD	Simvastatin, 40 QD for 4 weeks, then 80 QD	Pravastatin, 40 QD
Duration of intervention		24 weeks	24 weeks	24 weeks	24 weeks followed by 24 weeks open-label treatment	24 weeks
End points		Primary: SPAP	Primary: 6MWD	Primary: 6MWD	Primary: RV mass by CMR	Primary: Exercise time at a symptom-limited Naughton exercise stress test
		Secondary: 6MWD, hemodynamic parameters	Secondary: time to clinical worsening, Borg dyspnea score and hemodynamic parameters	Secondary: Borg dyspnea score, laboratory tests (e.g. NT-proBNP, plasma-thromboglobulin)	Secondary: 6MWD, Borg dyspnea score, NT-proBNP etc.	Secondary: SPAP, Borg dyspnea score, etc.
Outcomes		A trend of lowering SPAH and improving 6MWD, but no statistical significance was detected	No significant difference	No significant effect on the 6MWD, although the 6MWD of patients from simvastatin group tended to be lower at 6 months	RV mass and NT-proBNP decreased in simvastatin group, but not sustained at 12months. No significant improvement of 6MWD.	Significant improvement in the exercise time and the Borg dyspnea score

**Abbreviations**: RCT, randomized controlled trial; PH, pulmonary hypertension; COPD, chronic obstructive pulmonary diseases; CTEPH, chronic thromboembolic pulmonary hypertension; BID, twice daily; QD, daily; SPAP, systolic pulmonary arterial pressure; 6MWD, 6-minute walk distance; NT-proBNP, N-terminal pro–B-type natriuretic peptide; RV, right ventricle; CMR, cardiac magnetic resonance; SPAH, systolic pulmonary arterial hypertension.

### Primary outcome

**Fig 2:**
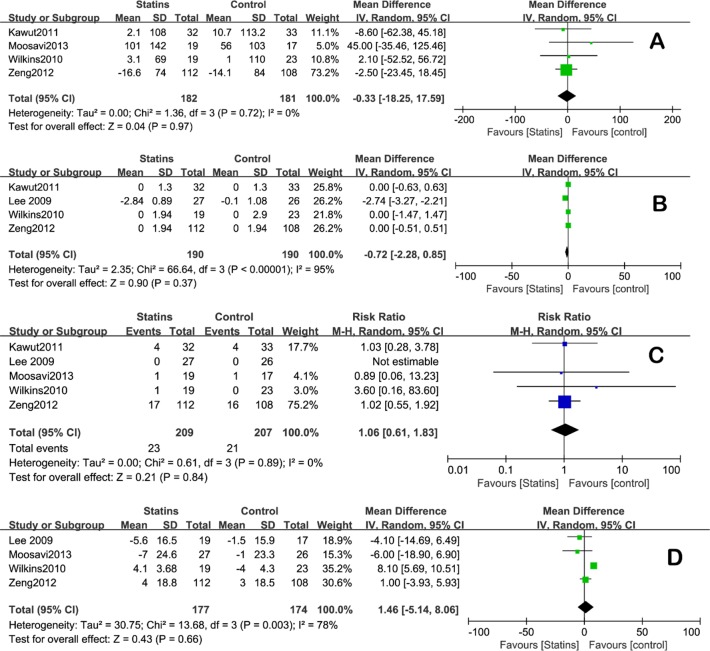
**Forest plot comparing statins group with control group** for A) 6MWD, B) Borg dyspnea score, C) clinical worsening risk ratio, and D) SPAP.

4 of the 5 included trials reported the change of 6MWD from baseline to week 24 [12–14, 17]. Statins administration didn’t affect 6MWD significantly as compared to the control group (mean difference [MD] = -0.33 [CI: -18.25 to 17.59], Fig 2A). There was no statistical heterogeneity among studies (I^2^ = 0). Lee’s study was not included in this analysis because they adopted a symptom-limited Naughton exercise stress test other than 6MWD as the measure for exercise capacity, and the result from it would be addressed in the Discussion section in a narrative way.

### Secondary outcomes

#### Borg dyspnea score

Four studies included Borg dyspnea score in their results [13, 14, 17, 19]. The analysis showed that statins didn’t decrease the score significantly, which was used as an index to the exercise capacity and quality of life (MD = -0.72 [CI: -2.28 to 0.85], Fig 2B). There was a statistical heterogeneity among studies (I^2^ = 95%). Sensitivity analysis was carried out and showed that the pooled effect would not be changed by omitting Lee’s study but the heterogeneity would become acceptable (heterogeneity: I^2^ = 0%).

#### Clinical worsening

Rate of clinical worsening was reported or could be calculated in all five studies; overall, the rate was 11.0% in the statins group as compared to 10.1% in the controls group. No significant difference was found (Risk ratio = 1.06 [CI: 0.61, 1.83], I^2^ = 0, Fig 2C).

#### SPAP

Only two studies measured the change of SPAP before and after statins administration and compared to controls [12, 19]. As a widely accepted method, we use the right ventricle systolic pressure (RVSP) from the other two articles as a substitute for SPAP [24], while the last article didn’t specify either of them. The result showed that statins didn’t affect SPAP significantly (MD = 1.46 [CI: -5.14, 8.06], Fig 2D). There was a statistical heterogeneity among studies (I^2^ = 78%). Sensitivity analysis was performed and showed that the pooled effect would not be changed by omitting Wilkins’s study but the heterogeneity would become acceptable (I^2^ = 0%).

### Subgroup analysis

#### PH due to non-COPD

**Fig 3:**
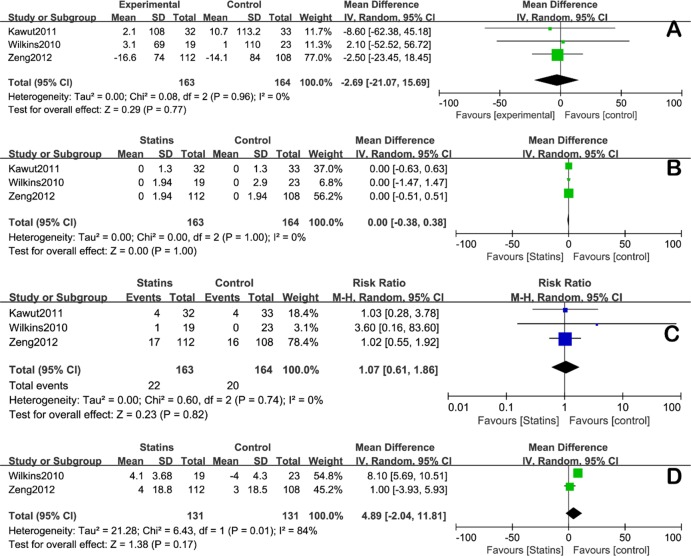
**Forest plot of subgroup analysis on non-COPD PH** for A) 6MWD, B) Borg dyspnea score, C) clinical worsening risk ratio, and D) SPAP.

**Fig 4:**
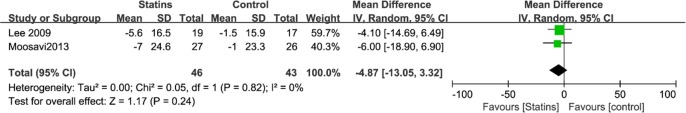
Forest plot for SPAP: subgroup analysis on PH due to COPD

Analysis of 327 patients suffering from PH due to non-COPD causes showed that, statins have no additional impact on: 1) change of 6MWD (MD = -2.69 [CI: -21.07, 15.69], Fig 3A), 2) Borg dyspnea score (MD = 0.00 [CI: -0.38, 0.38], Fig 3B), 3) rate of clinical worsening (RR = 1.07 [CI: 0.61, 1.86], Fig 3C) and 4) SPAP (MD = 4.89 [CI: -2.04, 11.81], Fig 3D)(for which RVSP was used as a substitute and only 262 patients were included due to lack of data).

#### PH due to COPD

The two studies involving PH due to COPD [12, 19] used different parameters as endpoints from each other, leaving SPAP as the only data for meta-analysis, which showed a trend to decrease but no significant difference between statins and control groups (MD = -4.87 [CI: -13.05 to 3.32], Fig 4).

Exercise capacity was increased by statins, although which wasn’t analyzed in this subgroup because the only two relevant studies had adopted different tools to examine the exercise capacity [12, 19]. To be specific, the Moosavi’s study showed that the difference in 6MWD at week 24 between atorvastatin and control group was in favor of atorvastatin (from 238 ± 124 to 339 ± 155 m, P = 0.003), but the change from baseline was not statistically significant compared to control [12]. Meanwhile the Lee’s study used a symptom-limited Naughton exercise stress test, and after 24 weeks, the exercise time of pravastatin group significantly increased 52% (from 660±352 s to 1006±316 s, *P <* 0.0001), which was also significantly better than control group [19]. Those results suggested that the pooled data might have been favorable for statins if they were measured in unified method.

## Discussion

To investigate the role of statins in PH treatment, our meta-analysis incorporated 425 patients from 5 randomized controlled trials and showed that, compared with standard therapy alone, addition of statins to standard therapy neither significantly increase exercise tolerance (i.e. 6MWD) nor decrease the dyspnea extent (i.e. Borg dyspnea score) or SPAP. Furthermore, the clinical worsening risks between these two groups were similar. These results of our analysis suggested that, despite the transient impact on hemodynamic parameters and biomarkers reported by earlier studies [16, 18, 20, 25], statins showed no beneficial effect on the clinical course of PH.

Besides the ability to increase circulating vasorelaxant factors, the early studies of animal models considered the main mechanism of action as inhibiting cell proliferation and promoting vascular apoptosis [7, 11, 26]. However, structural changes may not be able to exert measurable effects on exercise tolerance or hemodynamic parameters in a short term as 24 weeks. Moreover, most of our included trials used the standard therapy as control, including diuretics, digoxin, bosentan, calcium channel blockers, sildenafil, and prostacyclin analogues, which might overlap with statins in terms of mechanism of action and make the benefit of statins indistinguishable. Finally, all of these 5 studies enrolled chronic and stable patients, who had been stabilized on previous treatment and might not response promptly to the addition of statins.

Worthy of note, however, the subgroup of PH due to COPD showed a trend of lowering SPAP by statins which wasn’t significant probably due to inadequate sample size. More important and intriguing result was the beneficial impact of statins on exercise capacity of PH patients due to COPD. Unfortunately, the study by Lee [19] used a symptom-limited Naughton exercise stress test to evaluate exercise capacity other than 6MWD, and reported significant improvement of excise time after pravastatin. This result couldn’t be integrated with Moosavi’s Study [12] which used 6MWD and also showed benefit from statins; otherwise, it might have led to a beneficial difference in the subgroup analysis with COPD patients.

Those potential benefits may result from the mechanism of PH caused by COPD, which involves two important neurohormonal factors: IL-6 and ET-1. IL-6 mediates exaggerated inflammatory response induced by smoking and oxidative stress, and a correlation was found between SPAP and IL-6 [27, 28]. Statins can lower serum IL-6 through inhibition of guanosine triphosphatases (GTPases), which may reduce the inflammatory reaction. ET-1 causes vasoconstriction and vascular cell proliferation. In patients of idiopathic PAH, plasma levels of ET-1 correlate with pulmonary vascular resistance and inversely with prognosis [29]. Elevated plasma ET-1 levels are also detected in COPD patients [30]. Statins are related to the inhibition of synthesis of small GTP-binding proteins required for the activation of RhoA, which plays an important role in the sustained vasoconstriction mediated by ET-1. In Lee’s study, the encouraging results may be attributed to the effect of inhibiting ET-1 synthesis by pravastatin in PH patients with COPD [19]. Besides, oxidative stress increases in COPD patients and free radicals contribute to its pathophysiology [31, 32]. Statins can attenuate free radical generation [33–35], which may explain the effects on pulmonary vascular remodeling.

In short, the beneficial results of statins on PH due to COPD are not conclusive and the mechanisms remain unclear. It’s worthy of further investigation. We recommend the investigators explore the effect of statins on those patients in larger sample and longer follow-up, along with the relationships between the effect and the changes of IL-6 and ET-1 levels.

Last but not least, it is worth of noting that, in contrast with the trend to decrease in SPAP in statins group, there was a trend to increase in SPAP in non-COPD patients treated with statins (Fig 3D), which might be due to the heterogeneity of causes and mechanisms of PH. This result was important to recognize because it implied that statins might be harmful to non-COPD patients of PH.

## Conclusion

The pooled analysis of 5 RCTs done in PH patients shows that statins have no additional beneficial effect on standard therapy for PH, but the results from a subgroup of PH due to COPD seem intriguing and further study with larger sample size and longer follow-up is suggested.

## Supporting Information

S1 File PRISMA 2009 flow diagram. (DOC)

S2 File PRISMA 2009 checklist. (DOC)
