# Supplementary material for: Statins Have No Additional Benefit for Pulmonary Hypertension: A Meta-Analysis of Randomized Controlled Trials
Source: PLoS One. 2016 Dec 19;11(12):e0168101. doi: 10.1371/journal.pone.0168101 (PMC5167271; doi:10.1371/journal.pone.0168101)
Supplement: S1 File — (DOC) [file pone.0168101.s001.doc]

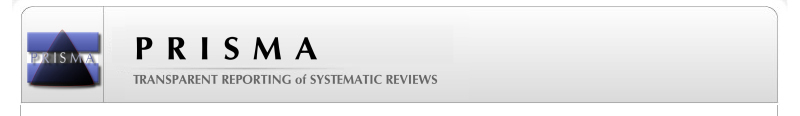
**PRISMA 2009 Flow Diagram**

**Screening**

**Included**

**Eligibility**

**Identification**

Records identified through database searching
(n = 165 )

Additional records identified through other sources
(n = 0 )

Records after duplicates removed
(n = 135 )

Records screened
(n = 135 )

123 Records excluded, because they were:

-biomarker study (n=4),

-animal study (n=14),

-registries (n=4),

-study design (n=1),

-observational study (n=15),

-non-relevant (n=85).

Full-text articles assessed for eligibility
(n = 12 )

Full-text articles excluded, because they didn’t meet the inclusion criteria (n = 7 )

Studies included in qualitative synthesis
(n = 5 )

Studies included in quantitative synthesis (meta-analysis)
(n = 5 )
